# Carotid Artery Disease in Subjects with Type 2 Diabetes: Risk Factors and Biomarkers

**DOI:** 10.3390/jcm11010072

**Published:** 2021-12-24

**Authors:** Vadim V. Klimontov, Elena A. Koroleva, Rustam S. Khapaev, Anton I. Korbut, Alexander P. Lykov

**Affiliations:** Research Institute of Clinical and Experimental Lymphology—Branch of the Institute of Cytology and Genetics, Siberian Branch of Russian Academy of Sciences (RICEL—Branch of IC&G SB RAS), 630060 Novosibirsk, Russia; ekoro@bk.ru (E.A.K.); khapaev.r.s@niikel.ru (R.S.K.); anton.korbut@gmail.com (A.I.K.); aplykov2@mail.ru (A.P.L.)

**Keywords:** atherosclerosis, carotid arteries, carotid stenosis, diabetes, risk factors, biomarkers

## Abstract

Carotid atherosclerosis (CA) and, especially, carotid artery stenosis (CAS), are associated with a high risk of cardiovascular events in subjects with type 2 diabetes (T2D). In this study, we aimed to identify risk factors and biomarkers of subclinical CA and CAS in T2D individuals. High-resolution ultrasonography of carotid arteries was performed in 389 patients. Ninety-five clinical parameters were evaluated, including diabetic complications and comorbidities; antihyperglycemic, hypolipidemic, and antihypertensive therapy; indices of glycemic control and glucose variability (GV); lipid panels; estimated glomerular filtration rate (eGFR); albuminuria; blood cell count; and coagulation. Additionally, serum levels of calponin-1, relaxin, L-citrulline, and matrix metalloproteinase-2 and -3 (MMP-2, -3) were measured by ELISA. In univariate analysis, older age, male sex, diabetes duration, GV, diabetic retinopathy, chronic kidney disease, coronary artery disease, peripheral artery disease, and MMP-3 were associated with subclinical CA. In addition to these factors, long-term arterial hypertension, high daily insulin doses, eGFR, and L-citrulline were associated with CAS. In multivariate logistic regression, age, male sex, BMI, GV, and eGFR predicted CA independently; male sex, BMI, diabetes duration, eGFR, and L-citrulline were predictors of CAS. These results can be used to develop screening and prevention programs for CA and CAS in T2D subjects.

## 1. Introduction

Cardiovascular diseases are the leading cause of death globally. According to the World Health Organization, 17.9 million people died from cardiovascular diseases (CVDs) in 2019, representing 32% of all global deaths; most of these deaths were due to heart attack and stroke [1]. Therefore, elaboration of predictive models of cardiovascular diseases remains a priority for clinical medicine. A number of epidemiological studies have revealed increased carotid intima–media thickness (CIMT) as a risk factor for myocardial infarction and stroke [2]. Identification of carotid plaques, compared with CIMT, further increases predictive accuracy for myocardial infarction [3]. Extracranial internal carotid artery stenosis (CAS) is a major cause of ischemic stroke, and it is estimated to cause 8–15% of such events [4].

A strong association between the presence of carotid atherosclerosis (CA) and cardiovascular events has been shown in individuals with diabetes [5,6]. It was revealed that subjects with diabetes and asymptomatic CAS have an extremely high risk of cardiovascular death. In a recent prospective study by M. Hoke et al., only 21% of the diabetic patients with > 50% asymptomatic carotid narrowing survived for a median of 11.8 years of follow-up [7].

It has been documented that the risk of CA is higher in diabetic patients than in the general population [8,9]. Accelerated CA in diabetes is usually attributed to the cumulative effect of traditional risk factors, such as age, smoking, hypertension, visceral obesity, dyslipidemia, and chronic kidney disease (CKD), although the contribution of each factor varies between studies [10,11,12,13,14]. Furthermore, the role of hyperglycemia as a promoter of CA has also been postulated [15,16]. A number of biomarkers—including inflammatory mediators, lipid and lipoprotein components, growth factors, adipokines, and microRNAs—have been proposed to improve the efficacy of asymptomatic CA screening and the prediction of carotid plaque vulnerability [17,18]. However, the superiority of these biomarkers over clinical risk factors remains ambiguous.

In this study, we assessed a wide range of clinical and laboratory parameters, as well as some regulatory molecules involved in vascular remodeling and atherogenesis, in order to identify risk factors and potential biomarkers of CA and CAS in subjects with type 2 diabetes (T2D).

## 2. Materials and Methods

### 2.1. Design 

An observational, single-center, cross-sectional study was conducted. Caucasian men and women aged at least 30 years with a known T2D duration of at least 1 year were included. A symptomatic carotid artery disease that requires revascularization therapy, bilateral carotid occlusions, stent implantation, or carotid endarterectomy in the medical history, current diabetic ketoacidosis or hyperglycemic hyperosmolar state, end-stage renal disease, malignant neoplasms, chronic inflammatory or autoimmune diseases, and acute infectious diseases within the previous 3 months were considered as exclusion criteria. Patients were recruited from January 2017 to December 2019 at the clinic of RICEL—Branch of IC&G SB RAS, a tertiary referral hospital.

A total of 389 patients were included in the study; all of them underwent a detailed clinical examination with assessment of diabetes control and in-depth screening/monitoring of diabetic complications and associated diseases. 

Ultrasound investigation of carotid arteries with estimation of CIMT and identification of plaques and CAS was performed. Based on the ultrasound results, subjects were divided into three groups: individuals with normal CIMT and no detectable plaques were defined as CA− patients; those with increased CIMT and/or plaque(s) but without hemodynamically significant stenosis were assigned to the CA+ CAS− group; individuals with significant CAS comprised the third group. 

Ninety-five clinical and laboratory parameters were evaluated as potential risk factors for CA and CAS (Table S1); among them, age; sex; smoking status; diabetes duration; body mass index (BMI); waist-to-hip ratio (WHR); diabetic complications and diabetes-related diseases; antihyperglycemic, hypolipidemic, and antihypertensive therapy; indices of glycemic control and glucose variability; lipid panels; renal function; albuminuria; blood cell count; and coagulation parameters were assessed.

In a sub-study of biomarkers we investigated the serum levels of calponin-1, relaxin-2, L-citrulline, matrix metalloproteinase-2 (MMP-2), and matrix metalloproteinases-3 (MMP-3). Among these molecules, calponin-1 is a marker of smooth muscle cell integrity [19], relaxin is an essential regulator of vascular tone [20], L-citrulline is involved in nitric oxide (NO) production and nitrogen stress [21], and MMP-2 and MMP-3 are important players in vascular remodeling, atherogenesis, and plaque instability [22]. The biomarker assay was performed in a sample of 152 patients; at least 40 people from each group of patients with diabetes were selected. A control group comprised 30 non-diabetic subjects with no cardiovascular risk factors in their medical history and no ultrasound signs of CA.

### 2.2. Methods

#### 2.2.1. Assessment of Clinical Risk Factors

Diabetes was diagnosed according to the World Health Organization criteria [23,24]. The diagnosis of chronic kidney disease (CKD) was based on the repeated measurements of albumin-to-creatinine ratio (ACR) and estimated glomerular filtration rate (eGFR) in accordance with the Kidney Disease: Improving Global Outcomes (KDIGO) clinical practice guidelines [25]. Diabetic retinopathy was revealed by an ophthalmologist using dilated and comprehensive eye examination. Coronary artery disease (CAD) was recorded if the patient had a history of myocardial infarction, angina, or silent myocardial ischemia verified by invasive testing. Any kind of surgical coronary revascularization was recorded as revascularization surgery in medical history. Peripheral artery disease (PAD) was screened by the ankle–brachial index; the diagnosis was verified by peripheral angiography and/or duplex ultrasound. Both ischemic and hemorrhagic stroke were recorded as stroke events. Non-alcoholic fatty liver disease (NAFLD) was diagnosed by liver ultrasound and transient elastography according to the European Association for the Study of the Liver (EASL), European Association for the Study of Diabetes (EASD), and European Association for the Study of Obesity (EASO) guidelines [26].

#### 2.2.2. Ultrasonography of Carotid Arteries

Ultrasound investigation of the carotid arteries was performed by a licensed specialist with the use of a high-resolution color ultrasound system (Vivid 7™ Dimension with linear detector 7–12 MHz, GE Healthcare, Chicago, IL, USA). Scanning was carried out bilaterally in more than three different longitudinal projections, as well as transverse projections. 

The CIMT measurements were performed according to the Mannheim IMT Consensus, 2012 [27]. The CIMT was measured as an average of the segmental maximum along the distal 1 cm region of each common carotid artery, within a region free of plaques with a clearly identified double-line pattern. CIMT values above 0.9 mm were considered as increased. A plaque was identified as a focal wall thickening at least 50% greater than that of the surrounding vessel wall, or as a focal region with CIMT > 1.5 mm that protrudes into the lumen and is distinct from the adjacent boundary, according to the recommendations of the American Society of Echocardiography [28]. CAS was defined as at least 50% carotid artery narrowing, as carotid narrowing less than 50% is generally considered hemodynamically insignificant [29,30].

#### 2.2.3. Laboratory Investigations

HbA1c levels were measured by turbidimetric immunoinhibition with the use of an AU480 Chemical Analyzer (Beckman Coulter, Brea, CA, USA). Three fasting and three 2-h postprandial blood glucose values were obtained daily from each patient in a three-day series. The measurements were performed with a OneTouch Verio^®^ glucose meter (Johnson & Johnson/LifeScan, Milpitas, CA, USA). Based on the obtained values, mean glucose, mean amplitude of glycemic excursions (MAGE), low blood glucose index (LBGI), and high blood glucose index (HBGI) were estimated with the use of the EasyGV calculator (version 9.0.R2) [31].

Biochemical parameters—including serum glucose, total cholesterol, low-density lipoprotein (LDL) cholesterol, high-density lipoprotein (HDL) cholesterol, triglycerides, creatinine, and uric acid, as well as urinary creatinine—were measured using an AU480 Chemical Analyzer (Beckman Coulter, USA) and commercially available cartridges. The estimated glomerular filtration rate (eGFR) was calculated by the CKD-EPI formula (2009). Urinary albumin was determined by immunoturbidimetry with an AU480 Chemical Analyzer (Beckman Coulter, USA). Complete blood counts were performed using a BC-5300 hematology analyzer (Mindray Medical International Limited, Shenzhen, China). Plasma concentrations of fibrinogen, soluble fibrin monomer complex (SFMC), and D-dimer were evaluated using an ACL Elite Pro automatic hemostasis analyzer (Instrumentation Laboratory, Bedford, MA, USA). 

The blood samples for the biomarker investigation were obtained from the cubital vein in the fasting state. Sera were separated and stored at −80 °C until analysis. The levels of calponin-1, relaxin, L-citrulline, MMP-2, and MMP-3 were assessed by ELISA with the use of an Infinite^®^ F50 microplate reader (Tecan, Männedorf, Switzerland) and commercially available kits (CUSABIO Technology LLC, Houston, TX, USA, for calponin-1; Immundiagnostik AG, Bensheim, Germany, for relaxin and L-citrulline; Abcam, Cambridge, UK, for MMP-2; BCM Diagnostics, Wilmington, DE, for MMP-3).

### 2.3. Statistical Analysis

The Statistica 13.0 software package (Dell, Round Rock, TX, USA) was used for most of the applied statistical procedures. The sample size was calculated with a predetermined type I error rate of α = 0.05 and a power goal of 1–β = 90%. Quantitative data are presented as medians and lower and upper quartiles; frequencies are expressed as percentages (%). The Kolmogorov–Smirnov (KS) test was applied to test the normality. Student’s *t*-test or analysis of variance (ANOVA) was used to compare two or multiple groups, respectively, if quantitative parameters were distributed normally; otherwise, the non-parametric Mann–Whitney U-test or Kruskal–Wallis H-test were applied. The differences in categorical parameters were assessed using the χ^2^ test. *p*-Values below 0.05 were considered significant. Spearman’s rank correlation analysis was applied to test the association between variables. 

To assess the risk factors of CA, receiver operating characteristic (ROC) curve analysis was performed with the IBM SPSS Statistics 26.0 software package (IBM, Armonk, NY, USA). The area under the ROC curve (AUC) with 95% confidence intervals (CI) and *p*-values was calculated. The results were considered significant if the AUC with a lower border of 95% CI was above 0.5 and the *p*-value was below 0.05. The cutoff values were selected with both sensitivity (Se) and specificity (Sp) above 0.55. Non-normally distributed laboratory parameters (calponin-1, relaxin, L-citrulline, and MMP-2) were also assessed as decimal logarithms (Log) in this analysis.

Furthermore, the identified factors were included in a multiple logistic regression analysis with forward selection of variances. The values of biomarkers and key laboratory parameters as possible risk factors of CA and CAS were assessed with adjustment to demographic factors and clinical parameters. The models with fewer, non-correlated parameters, lower KS statistics *p*-values, and higher AUC, Se, and Sp were selected. Crude and adjusted odd ratios (ORs), 95% CIs, and *p*-values were calculated for parameters included in the models. The characteristics of the models (i.e., intercept, KS statistics *p*-value, Se, Sp) and the cutoff points of logistic function (L_P_) are presented.

## 3. Results

### 3.1. Clinical Characteristics 

A total of 277 females and 112 males with T2D, aged from 36 to 88 years (median 65 years), were included in the study. The diabetes duration varied from 1 to 45 years (median 12 years). Twenty-two subjects had normal BMI, 87 participants were overweight, and 280 subjects were obese. The median level of HbA1c was 8.2% (range: 4.9–15.8%). All patients received antihyperglycemic therapy, which included metformin (*n* = 272), sulfonylurea (*n* = 126), dipeptidyl peptidase-4 (DPP4) inhibitors (*n* = 41), sodium glucose cotransporter 2 (SGLT2) inhibitors (*n* = 38), glucagon-like peptide-1 (GLP-1) receptor agonists (*n* = 8), and insulin (*n* = 240)—mostly in combinations.

According to the ultrasonography results, 54 individuals had no signs of CA (CA− group), 201 demonstrated increased CIMT and/or plaques in carotid arteries without ≥50% narrowing (CA+ CAS− group), and 134 subjects had CAS (CAS group).

As it shown in Table 1, CA was more common among men. Patients with CA, regardless of the presence of CAS, were older and less obese, had longer diabetes duration, and were more likely to have CKD, CAD, myocardial infarction, and PAD in their medical history. Among patients with CA, those with CAS were older, had longer diabetes and hypertension durations since diagnosis, and higher prevalence of diabetic retinopathy, CKD, CAD, PAD, myocardial infarction, and revascularization surgery in their medical history. Brain stroke was more frequent in patients with CAS compared to subjects with normal carotid arteries. Other studied parameters did not show significant differences between the groups. 

Antihyperglycemic treatment was similar in the studied groups. Among patients on insulin, the duration of insulin therapy was longer in those with CA and CAS. In these last two groups, antiplatelet drugs and statins were used more often.

### 3.2. Laboratory Parameters

The mean levels of HbA1c were highest in CA+ CAS− patients, though no significant differences were detected between the groups (Table 2). At the same time, MAGE and urinary albumin-to-creatinine ratio (UACR) values were significantly higher and eGFR was lower in subjects with CAS when compared to the CA− group. Lipid parameters, uric acid, and hematology parameters demonstrated no differences. The levels of fibrinogen and SFMC—but not D-dimer—were increased in patients with CAS when compared to patients without CA. Other laboratory parameters did not show significant differences. 

**Figure 1:**
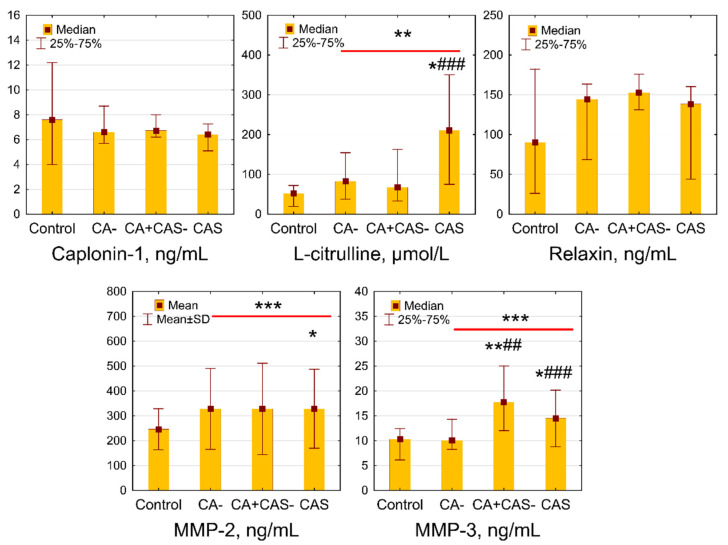
Serum concentrations of biomarkers in T2D patients, depending on the presence of CA and CAS. Normally distributed data (MMP-2) are presented as means ± SD; non-normally distributed data (others) are presented as medians (25th–75th percentiles). * *p* < 0.05, ** *p* < 0.01, *** *p* < 0.001 vs. CA− group; ## *p* < 0.01, ### *p* < 0.001 vs. controls. CA: carotid atherosclerosis; CAS: carotid artery stenosis; MMP-2: matrix metalloproteinase-2; MMP-3: matrix metalloproteinase-3; T2D: type 2 diabetes.

Patients with diabetes, as a whole group, demonstrated increased serum levels of L-citrulline (*p* = 0.006), MMP-2 (*p* = 0.0002), and MMP-3 (*p* = 0.008) as compared to controls. Calponin-1 and relaxin did not show significant differences (*p* = 0.59 and *p* = 0.44, respectively). The concentrations of L-citrulline were significantly higher in patients with CAS as compared to the control and CA− groups (Figure 1). The levels of MMP-3 were increased significantly in the CA+ CAS− and CAS groups when compared to control and CA− patients. Meanwhile, the serum concentrations of MMP-2 were elevated in the CAS group compared to controls. L-citrulline and MMP-3 demonstrated weak positive correlations with mean CIMT (r = 0.26, *p* = 0.001 and r = 0.18, *p* = 0.03, respectively). Moreover, L-citrulline showed a weak negative correlation with eGFR (r = −0.18, *p* = 0.03).

### 3.3. Risk Factors for CA and CAS in Univariate Analysis

The cutoff values for quantitative parameters associated with CA and CAS were estimated by the ROC curve analysis (Table 3). Ages of ≥62 and ≥66 years, BMI of ≤34.5 and ≤32.5 kg/m^2^, and diabetes durations of ≥11 and ≥13 years were revealed as risk factors for CA and CAS, respectively. In addition, MAGE ≥ 3.38 mmol/L and log MMP-3 ≥ 1.12 were associated with CA, while duration of hypertension ≥18 years, daily insulin dose ≥ 0.585 IU/kg, eGFR ≤ 65.5 mL/min/1.73 m^2^, log L-citrulline ≥ 2.1, and log MMP-3 ≥ 1.1 were identified as risk factors for CAS. Other studied parameters showed no significant associations.

In the next step, we estimated the odd ratios for the quantitative parameters revealed by ROC curve analysis and binary parameters showing the differences between the groups, considering these as the risk factors for CA and CAS (Table 4). 

In univariate analysis, age ≥ 62 years, myocardial infarction in medical history, and log MMP-3 ≥ 1.12 were associated with the highest increases in CA risk (OR = 4.7, OR = 4.47, and OR = 4.45, respectively). Male sex, BMI ≤ 34.5 kg/m^2^, diabetes duration ≥ 11 years, MAGE ≥ 3.38 mmol/L, and the presence of diabetic retinopathy, CKD, CAD, and PAD demonstrated ORs ranging from 1.97 to 3.68. 

Age, BMI ≤ 32.5 kg/m^2^, duration of diabetes and hypertension ≥13 years and ≥18 years, respectively, daily insulin dose ≥ 0.585 IU/kg, eGFR ≤ 65.5 mL/min/1.73 m^2^, log L-citrulline ≥ 2.1, and the presence of diabetic retinopathy, CKD, CAD, PAD, and myocardial infarction in medical history were associated with CAS. Among these factors, log L-citrulline, age, myocardial infarction, and CKD were the most reliable predictors.

### 3.4. Risk Factors for CA and CAS in Multivariate Analysis

In multivariate logistic regression analysis, age, BMI, male sex, MAGE, and eGFR were independent predictors of CA, while serum MMP-3 demonstrated borderline significance (Table 5). Male sex, BMI, diabetes duration, eGFR, and serum L-citrulline were associated with CAS independently after adjustment for other risk factors included in the model.

### 3.5. Combinations of the Risk Factors for CA and CAS

In the next step, we identified combinations of the factors associated with CA and CAS in order to improve predictive reliability (Table 6). Three combinations (“age ≥ 62 years AND male sex AND duration of diabetes ≥ 11 years”, “age ≥ 62 years AND male sex AND BMI ≤ 34.5 kg/m^2^”, and “age ≥ 62 years AND duration of diabetes ≥ 11 years AND MAGE ≥ 3.38 mmol/L”) provided the highest OR values when assessing the association with CA. These combinations were characterized by very high specificity but low sensitivity. A combination of age ≥ 62 years (or duration of diabetes ≥ 11 years) with macrovascular disease (CAD OR PAD) was the most sensitive. Among combinations associated with CAS, the combination of age ≥ 66 years and log L-citrulline ≥ 2.1 was the most specific, and provided the highest OR value. However, the sensitivity of this combination was low. 

## 4. Discussion

In this study, we identified the risk factors and assessed some potential biomarkers of CA and CAS in subjects with T2D. The development of CA in T2D subjects was determined by a combination of general and diabetes-related risk factors. 

### 4.1. General Risk Factors for CA and CAS in Subjects with T2D 

According to our data, age ≥ 62 years, male sex, and BMI ≤ 34.5 kg/m^2^ were associated with CA; meanwhile, age and BMI ≤ 32.5 kg/m^2^ were related to CAS. 

While the role of older age as a risk factor for CA is not questioned [14,32], the data on the relationship of CA with gender and body weight in patients with T2D are rather inconsistent. Some authors have identified male gender as a risk factor for CA and CAS in T2D subjects [10,13]; meanwhile, others have observed a higher prevalence of CA in T2D women [14,32]. In previous studies, visceral adiposity was linked to CA in subjects with T2D [14,33]. Conversely, in our cohort lower BMI was associated with CA and CAS in the ROC analysis. It should be noted that this association was not confirmed by logistic regression analysis after adjustment for age and other risk factors. Therefore, we cannot exclude the possibility that the association between BMI and CA is mediated by confounders. However, a recent study indicated that the risk of CA increases linearly with decreasing muscle mass in men and women with T2D [34]. The role of body composition parameters as risk factors for CA needs further research.

The results of previous research linked CA with dyslipidemia [11,13]. In our patients, no connection was found between the levels of lipids and CA. This fact can be explained by more intensive hypolipidemic therapy with statins in the atherosclerosis groups. A long history of arterial hypertension (≥18 years) was associated with CAS, which is consistent with the data of other authors [10,11,13]. At the same time, we did not detect the significance of smoking among the risk factors, as was noted previously [14]. Unfortunately, we did not take into account previous smoking habits and smoking intensity.

Our results do not confirm the value of the neutrophil-to-lymphocyte ratio as a risk factor for CAS—as has been reported recently [35]. Although the levels of fibrinogen and SFMC were higher in patients with CAS, these parameters did not show significance in the ROC analysis and multivariate regression.

### 4.2. Diabetes-Related Risk Factors for CA and CAS 

The role of hyperglycemia as a promoter of atherogenesis is discussed widely [36,37]. In mixed diabetic and non-diabetic populations, glycemic status has been recognized as a risk factor for CA [15] and CAS [13,15,16,38]. In our cohort, diabetes duration ≥ 11 and ≥13 years was associated with CA and CAS, respectively, in the ROC analysis; moreover, diabetes duration was an independent risk factor for CAS in the multivariate model.

Although we did not find any association of CA and CAS with HbA1c levels, we identified increased MAGE (≥3.38 mmol/L)—an indicator of glucose variability—as a risk factor. This finding is consistent with recently published data demonstrating an association between the grayscale median of the carotid arteries and glucose variability parameters in Japanese patients with T2D [39]. Another study recognized standard deviation, coefficient of variation, and MAGE—derived from continuous glucose monitoring—as independent factors associated with severe intracranial carotid artery siphon stenosis in T2D subjects [40]. The association between CA and 1,5-anhydroglucitol—an intermediate-term indicator of glucose variability—has also been reported [41]. The deteriorating effect of glucose variability on the vascular wall can be observed in the form of oxidative stress, non-enzymatic glycation, chronic inflammation, endothelial dysfunction, platelet activation, impaired angiogenesis and renal fibrosis [42,43].

When analyzing the antihyperglycemic treatment, we noted the longer duration of insulin therapy in patients with CA and CAS. In ROC analysis, we identified higher daily insulin dose (≥0.59 IU/kg) as a factor associated with CAS. The relationship of insulin therapy with the risk of atherosclerosis in T2D patients is still controversial. It is well known that insulin treatment can increase glucose variability—a direct relationship between glucose variability and insulin dose has been reported [44]. Other potentially atherogenic adverse effects of high doses of insulin in T2D include weight gain, recurrent hypoglycemia, and iatrogenic hyperinsulinemia [45]. A recent retrospective analysis of a large cohort of T2D subjects has shown that insulin therapy is associated with a markedly increased risk of CA; this effect was partly attributed to insulin resistance [46].

In our patient cohort, CA was related to both microvascular and macrovascular disease. Specifically, diabetic retinopathy, CKD, CAD (including myocardial infarction in anamnesis), and PAD were more prevalent in those with CA. In addition, the prevalence of myocardial revascularization surgery and stroke was higher in the group with advanced atherosclerosis estimated by the presence of CAS. Among diabetic complications, diabetic kidney disease is considered to be a promoter of atherosclerosis progression [11,14]. Disorders of the mineral metabolism, such as higher phosphate and lower 25(OH) vitamin D levels, as well as vascular calcification, lipid profile abnormalities, hyperuricemia, anemia, inflammation, cell senescence, oxidative stress, endothelial dysfunction, and uremic toxins, are considered to be contributors to accelerated atherogenesis in CKD [47]. Accordingly, we identified eGFR ≤ 65.5 mL/min/1.73 m^2^ as a risk factor for CAS in T2D subjects.

### 4.3. The Search for Biomarkers of CA and CAS in Subjects with T2D 

In addition to clinical risk factors, in this study we assessed the value of some molecules involved in vascular remodeling and atherogenesis (i.e., calponin-1, relaxin, L-citrulline, MMP-2, and MMP-3) as potential biomarkers of CA and CAS in T2D subjects. The choice of these regulators was based on their physiological effects. Calponin-1—a thin-filament-associated protein—plays a role in smooth muscle contractility, and is expressed specifically in smooth muscle cells [19]. Relaxin—a peptide hormone—modulates systemic and renal vascular tone [20]. Additionally, relaxin demonstrates antifibrotic properties, inhibiting fibroblast proliferation and/or differentiation into myofibroblasts, suppressing collagen synthesis, and promoting the expression and activity of MMPs [48]. L-citrulline is a non-essential alpha-amino acid with antioxidant and vasodilation properties; it belongs to the NO system [21]. The collagen-degrading enzymes MMP-2 and MMP-3 are involved in vascular remodeling, reparation, and angiogenesis [22].

The levels of calponin-1 and relaxin were not significantly altered in subjects with T2D, and demonstrated no association with CA. Experimental data on the expression of calponin in smooth muscle cells under hyperglycemic conditions are contradictory. It has been reported that high extracellular glucose enhances the expression of calponin in smooth muscle cells in vitro [49]. In Goto–Kakizaki rats—a model of non-obese T2D—the expression of calponin in the aorta was also increased [50]. However, other experimental data indicate that cyclic strain and low wall shear stress under chronic exposure to high glucose reduce the expression of calponin in vascular smooth muscle cells [51].

Exogenous relaxin administration has demonstrated significant therapeutic benefits in animal models of hypertension and atherosclerosis, and is considered to be a potential therapeutic target in cardiovascular complications of diabetes. Relaxin upregulates NO production in the vasculature and improves endothelial function under hyperglycemic conditions [52]. Previous studies revealed decreased serum levels of relaxin in patients with T2D, with inconsistent data on the relationship with glycemic control [53,54]. It was reported that in subjects with CA and PAD, relaxin expression in the arterial walls, as well as serum relaxin levels, is inversely associated with the severity of atherosclerotic lesions [55,56]. Nevertheless, in our patients, we found no association between relaxin and CA. Some confounders are likely to modify the link between relaxin and atherosclerosis in diabetes. Specifically, in women with newly diagnosed T2D, plasma relaxin levels were positively related to insulin sensitivity [57]. It should be noted that relaxin is not a specific cardiovascular biomarker. In particular, the ovaries and prostate are important sources of circulating relaxin [52].

According to the obtained results, patients with T2D have increased serum levels of L-citrulline, MMP-2, and MMP-3, wherein L-citrulline and MMP-3 demonstrate an association with CAS and CA, respectively.

In accordance with our data, two previous studies documented increased serum levels of L-citrulline in subjects with T2D [58,59]. In these studies, L-citrulline concentrations were correlated positively with HbA1c. In mice, plasma L-citrulline levels increased when animals became obese on a high-fat diet, demonstrating associations with hyperglycemia and NAFLD [60]. The nature of the increased L-citrulline levels in T2D is not entirely clear. It is known that L-citrulline is produced from L-arginine by NO synthases, alongside NO. Moreover, L-citrulline is released into the blood stream from the small intestine, where it is synthesized de novo from arginine or glutamine. L-citrulline is also a product of the urea cycle in the liver [60]. Since L-citrulline is metabolized primarily in the kidneys, its levels increase as renal function declines [61]. In our work, we found a weak negative correlation between L-citrulline concentration and eGFR. Thus, it is possible to assume a variety of mechanisms for the increase in the levels of L-citrulline in T2D. 

Some studies have revealed a link between L-citrulline metabolism and diabetic vascular complications. It was found that dysregulation of citrulline-related pathways was related to diabetic retinopathy in subjects with T2D [62]. Magnusson M. et al. reported that higher levels of L-citrulline in subjects with no CVD at baseline were associated with cardiovascular risk during 12 years of follow-up; L-citrulline was also cross-sectionally related to CAS [63]. It was shown that the disturbances of the L-arginine-NO-L-citrulline pathway in diabetes and metabolic syndrome are closely related to inflammation and oxidative stress [64]—the hallmarks of diabetic vascular complications [36]. At the same time, it was demonstrated that L-citrulline supplementation increases NO levels [65], improves insulin resistance, and decreases HbA1c and concentrations of inflammatory markers in patients with T2D [66,67]. The abnormalities of L-citrulline metabolism in diabetes, along with the mechanisms of their relationship with vascular disease, merit further research.

MMPs are considered to be major drivers of vascular remodeling. Hemodynamic changes, vascular injury, inflammation, oxidative stress, and NO have strong impacts on MMP expression and activity [68,69,70]. At the same time, the shifts in the activity of MMPs are important for vascular extracellular matrix alterations and atherosclerotic plaque neovascularization and vulnerability. MMP-3 is of particular interest among these enzymes, as it is not produced by healthy vessels, but is naturally present in atherosclerotic plaques, expressed in activated vascular smooth muscle cells, macrophages, and lymphocytes [22]. Experimental data indicate that MMP-3 is involved in the intimal hyperplasia in a carotid artery ligation model, and promotes migration of vascular smooth muscle cells [71]. It was suggested that MMP-3 could be beneficial for plaque stabilization by promoting fibrous cap formation [72]. Conversely, in patients with CA, serum MMP-3 protein levels were increased in those with vulnerable plaques, and were positively correlated with a 2-year occurrence of cerebrovascular events [73]. A correlation between MMP-3 activity and CIMT was found in untreated subjects with arterial hypertension [74]. In the general population, active and total MMP-3 demonstrated a relationship with the carotid plaque score [75]. It was reported that single-nucleotide polymorphism (rs3025058) in the MMP-3 gene is associated with CA in the general population [76] and in subjects with T2D [77].

Our study did not reveal any significant differences between the control group and patients with diabetes. This may be because hyperglycemia in patients with diabetes mellitus can lead to the simultaneous activation of not only a contractile gene program—manifested by vasospasm and hypertension of resistant vessels—but also a synthetic program leading to an increased risk of neointimal hyperplasia and stenosis. The extent to which these gene programs can be activated can also vary depending on the vascular bed (i.e., the dominance of the synthetic program in regions prone to atherosclerosis or contractile predominance of resistant arteries).

### 4.4. Limitations and Future Remarks 

A limitation of our study is its cross-sectional design, which does not prove causality. The recruitment of patients at one clinical center could lead to some sampling bias. Not all participants were tested for biomarkers. While studying the molecules involved in the availability of NO, we did not evaluate NO synthesis per se. In addition, we did not measure the concentrations of metalloproteinases other than MMP-2 and MMP-3, nor those of metalloproteinase inhibitors.

At the same time, this is the first study to assess a wide range of demographic, anthropometric, clinical, and laboratory parameters, including some biomarkers, to individualize the risk assessment of CA and CAS in T2D subjects. It should be noted that the changes in the carotid arteries can be partially reversible. It has been demonstrated that intensification of risk factor intervention in T2D subjects results in CIMT regression over a period of 2 years [78]. Therefore, our risk factor profile data can be used to design a secondary prevention strategy for CA and CAS in T2D subjects.

The value of a number of identified factors and biomarkers of CA and CAS needs to be tested in prospective studies. Specifically, the significance of glucose variability and high-dose insulin therapy as possible risk factors for CA deserves special attention. The role of L-citrulline and MMP-3 in the pathophysiology of CA and CAS also merits further research.

## 5. Conclusions

In this study, we have demonstrated that in subjects with T2D, older age, male sex, diabetes duration, glucose variability, and the presence of microvascular (diabetic retinopathy, CKD) and macrovascular (CAD, PAD) disease are associated with subclinical CA. In addition to these factors, lower BMI, long-term arterial hypertension, high insulin dose, and reduced eGFR are associated with CAS. Serum MMP-3 and L-citrulline can be considered with some caution as biomarkers associated with CA and CAS, respectively, in subjects with T2D, though the predictive value of these molecules needs further research. 

## Figures and Tables

**Table 1:** Clinical characteristics of patients with T2D with and without CA and CAS.

Parameter	CA− *n* = 54	CA+ CAS− *n* = 201	CAS *n* = 134
**General parameters**			
Sex, m/f, *n* (%)	9/45 (16.7/83.3)	61/140 (30.3/69.7) *	42/92 (31.3/68.7) *
Age, years	58 (51–63)	64 (58–70) ***	68 (63–71) ***,###
Weight, kg	93.5 (83–120)	88 (76–102) *	86 (74–94) ***
BMI, kg/m^2^	36.8 (31.6–42.5)	32.9 (28.9–37.5) ***	31.9 (29.3–36.4) ***
Waist circumference, cm	115 ± 17	109 ± 13	112 ± 14
Hip circumference, cm	120 (105.5–130.5)	112 (100–120)	110 (104.5–117.5)
WHR	0.93 (0.91–1.01)	0.97 (0.91–1.04)	0.98 (0.94–1.07)
Current smoking, *n* (%)	7 (13.0)	17 (8.5)	20 (14.9)
Diabetes duration since diagnosis, years	8 (5–14)	12 (8–17) *	15 (8–22) ***,#
**Diabetes complications and associated diseases**			
Diabetic retinopathy, *n* (%)	20 (37.0)	95 (47.3)	85 (63.4) **,##
CKD, *n* (%)	34 (63.0)	156 (77.6) *	121 (90.3) ***,##
Hypertension, *n* (%)	50 (92.5)	195 (97.0)	132 (98.5) *
Hypertension duration, years	14 (9–20)	15 (8–22)	20 (10–30) *,##
CAD, *n* (%)	9 (16.6)	67 (33.3) *	75 (56.0) ***,###
Myocardial infarction, *n* (%)	2 (3.7)	19 (9.5) *	30 (22.4) ***,###
PAD, *n* (%)	15 (27.7)	90 (44.8) *	90 (67.7) ***,###
Revascularization surgery, *n* (%)	2 (3.7)	21 (10.4)	30 (22.4) **,##
Stroke, *n* (%)	2 (3.7)	20 (9.95)	18 (13.4) *
NAFLD, *n* (%)	39 (72.2)	125 (63.2)	77 (57.5)
**Treatment**			
Metformin, *n* (%)	39 (72.2)	145 (72.1)	88 (65.7)
Sulfonylurea, *n* (%)	15 (27.8)	76 (37.8)	35 (26.1) #
DPP4 inhibitors, *n* (%)	4 (7.4)	23 (11.4)	14 (10.4)
SGLT2 inhibitors, *n* (%)	6 (11.1)	20 (10.0)	12 (9.0)
GLP-1 receptor agonists, *n* (%)	2 (3.7)	4 (2.0)	2 (1.5)
Insulin, *n* (%)	28 (51.9)	121 (60.2)	91 (67.9)
Duration of insulin therapy, years	2 (0.1–6)	5 (1–10) *	7 (3–11) ***
Daily insulin dose, IU/kg	0.5 (0.3–0.8)	0.5 (0.3–0.7)	0.6 (0.4–0.8)
RAS blockers, *n* (%)	41 (75.9)	160 (79.6)	105 (78.4)
Diuretics, *n* (%)	23 (42.5)	94 (46.7)	71 (53.0)
Beta blockers, *n* (%)	21 (38.9)	90 (44.8)	67 (50.0)
Calcium channel blockers, *n* (%)	16 (29.6)	65 (32.3)	56 (41.8)
Antiplatelet agents, *n* (%)	18 (33.3)	116 (57.7) **	93 (69.4) ***,#
Statins, *n* (%)	15 (27.8)	86 (42.8) *	72 (53.7) **

Non-normally distributed continuous data are presented as medians (25th–75th percentiles); normally distributed continuous data are presented as means ± standard deviations (SD). * *p* < 0.05, ** *p* < 0.01, *** *p* < 0.001 vs. CA− group; # *p* < 0.05, ## *p* < 0.01, ### *p* < 0.001 vs. CA+ CAS− group. BMI: body mass index; CA: carotid atherosclerosis; CAD: coronary artery disease; CAS: carotid artery stenosis; CKD: chronic kidney disease; DPP4: dipeptidyl peptidase 4; GLP-1: glucagon-like peptide-1; NAFLD: non-alcoholic fatty liver disease; PAD: peripheral artery disease; RAS: renin angiotensin system; SGLT2: sodium glucose cotransporter 2; T2D: type 2 diabetes; WHR: waist-to-hip ratio.

**Table 2:** Laboratory parameters in patients with T2D depending on the presence of CA and CAS.

Parameter	CA− *n* = 54	CA+ CAS− *n* = 201	CAS *n* = 134
**Biochemical parameters**			
HbA1c, %	7.38 (6.30–9.90)	8.36 (7.23–10.0)	8.17 (7.05–9.55)
MAGE, mmol/L	2.96 (2.22–4.08)	3.67 (2.6–4.77)	4.03 (2.73–5.25) ***
LBGI, a.u.	0.02 (0–0.39)	0.14 (0–0.94)	0.18 (0–1.15)
HBGI, a.u.	8.22 (2.12–15.5)	7.24 (3.71–14.6)	7.68 (4.03–15.4)
Total cholesterol, mmol/L	4.99 (4.4–5.97)	5.10 (4.30–6.06)	4.92 (4.12–6.10)
LDL cholesterol, mmol/L	3.23 (2.74–3.95)	3.21 (2.59–3.97)	3.14 (2.36–4.03)
HDL cholesterol, mmol/L	1.15 (0.98–1.39)	1.17 (0.98–1.40)	1.14 (0.97–1.33)
Triglycerides, mmol/L	2.22 (1.62–3.14)	1.97 (1.30–2.96)	1.93 (1.30–2.71)
Uric acid, µmol/L	342 ± 86	326 ± 94	339 ± 94
**Renal tests**			
Serum creatinine, µmol/L	85.6 (73.0–95.3)	86 (74.2–97)	89.3 (77.0–106)
eGFR, mL/min/1.73 m^2^	69 (60–84)	68 (58–84)	62 (53–75) **,##
UACR, mg/mmol	0.95 (0.40–1.70)	1.05 (0.50–4.00)	1.50 (0.50–7.00) *
**Hematology and coagulation**			
Hemoglobin, g/L	137 (127–150)	138 (127–146)	137 (125–146)
RBCs, ×10^12^/L	4.67 (4.44–5.09)	4.69 (4.37–5.01)	4.64 (4.34–4.96)
WBCs, ×10^9^/L	6.63 (5.83–8.22)	6.52 (5.37–8.00)	6.70 (5.64–7.86)
Neutrophils, ×10^9^/L	3.82 (3.12–5.33)	3.96 (3.20–5.03)	3.96 (3.00–5.15)
Lymphocytes, ×10^9^/L	1.97 (1.64–2.44)	2.0 (1.64–2.43)	1.97 (1.57–2.52)
Neutrophil-to-lymphocyte ratio	1.98 (1.53–2.27)	1.96 (1.55–2.52)	2.11 (1.48–2.76)
Monocytes, ×10^9^/L	0.29 (0.20–0.37)	0.27 (0.21–0.36)	0.29 (0.21–0.38)
Eosinophils, ×10^9^/L	0.13 (0.10–0.20)	0.15 (0.11–0.22)	0.15 (0.09–0.21)
Platelets, ×10^9^/L	246 ± 63	246 ± 61	241 ± 60
Fibrinogen, mmol/L	4.0 (3.4–4.5)	4.3 (3.6–5.0)	4.3 (3.7–5.3) *
SFMC, mg/dL	8.0 (3.5–14.5)	10.0 (4.3–16.0)	13.0 (5.5–21.0) *
D-dimer, ng/mL	266 (228–349)	273 (237–321)	274 (247–330)

Non-normally distributed data are presented as medians (25th–75th percentiles); normally distributed data are presented as means ± standard deviations (SD). * *p* < 0.05, ** *p* < 0.01, *** *p* < 0.001 vs. CA− group. ## *p* < 0.01 vs. CA+ CAS− group. CA: carotid atherosclerosis; CAS: carotid artery stenosis; LBGI: low blood glucose index; eGFR: estimated glomerular filtration rate; HBGI: high blood glucose index; HDL: high-density lipoprotein; LBGI: low blood glucose index; LDL: low-density lipoprotein; MAGE: mean amplitude of glycemic excursions; RBCs: red blood cells; SFMC: soluble fibrin monomer complex; T2D: type 2 diabetes; UACR: urinary albumin-to-creatinine ratio; WBCs: white blood cells.

**Table 3:** ROC analysis of parameters associated with CA and CAS in subjects with T2D.

Parameter	Cutoff Point	AUC ± SE (95% CI), *p*-Value	Se	Sp	OR (95% CI), *p*-Value
**Parameters associated with CA**					
Age	≥62 years	0.761 ± 0.033, (0.696–0.826), *p* < 0.001	0.70	0.67	4.70 (2.55–8.67), *p* < 0.001
BMI	≤34.5 kg/m^2^	0.677 ± 0.040, (0.598–0.756), *p* < 0.001	0.61	0.61	2.47 (1.37–4.45), *p* = 0.003
Diabetes duration	≥11 years	0.649 ± 0.041, (0.570–0.729), *p* < 0.001	0.60	0.61	2.36 (1.31–4.25), *p* = 0.004
MAGE	≥3.38 mmol/L	0.619 ± 0.041, (0.538–0.700), *p* = 0.005	0.60	0.59	2.16 (1.20–3.87), *p* = 0.01
Log MMP-3	≥1.12	0.733 ± 0.063 (0.61–0.856), *p* = 0.001	0.69	0.70	4.45 (1.65–12.0), *p* = 0.003
**Parameters associated with CAS**					
Age	≥66 years	0.662 ± 0.028, (0.607–0.717), *p* < 0.001	0.62	0.62	2.61 (1.70–4.01), *p* < 0.001
BMI	≤32.5 kg/m^2^	0.571 ± 0.030, (0.512–0.629), *p* = 0.02	0.55	0.56	1.55 (1.02–2.37), *p* = 0.04
Diabetes duration	≥13 years	0.610 ± 0.030, (0.500–0.670), *p* < 0.001	0.59	0.56	1.83 (1.20–2.80), *p* = 0.005
Hypertension duration	≥18 years	0.616 ± 0.031, (0.555–0.677), *p* < 0.001	0.57	0.60	1.95 (1.26–3.04), *p* = 0.003
Daily insulin dose, IU/kg	≥0.59 IU/kg	0.580 ± 0.037, (0.508–0.651), *p* = 0.03	0.58	0.59	1.91 (1.14–3.20), *p* = 0.01
eGFR	≤65.5 mL/min/1.73 m^2^	0.608 ± 0.030, (0.548–0.667), *p* = 0.001	0.56	0.55	1.53 (1.003–2.33), *p* = 0.048
Log L-citrulline	≥2.10	0.675 ± 0.078 (0,523–0.827), *p* = 0.03	0.68	0.74	4.83 (1.47–15.9), *p* = 0.01
Log MMP-3	≥1.10	0.649 ± 0.054 (0.543–0.756), *p* = 0.01	0.63	0.65	2.78 (1.23–6.28), *p* = 0.01

AUC: area under the receiver operating characteristic curve; BMI: body mass index; CA: carotid atherosclerosis; CAS: carotid artery stenosis; CI: confidence interval; eGFR: estimated glomerular filtration rate; MAGE: mean amplitude of glycemic excursions; MMP-3: matrix metalloproteinase-3; OR: odds ratio; SE: standard error; Se: sensitivity; Sp: specificity; T2D: type 2 diabetes; UACR: urinary albumin-to-creatinine ratio.

**Table 4:** Parameters associated with CA and CAS in subjects with T2D in univariate analysis.

Parameter	OR	95% CI	*p*-Value
**Parameters associated with CA**			
Age ≥ 62 years	4.70	2.55–8.67	0.001
Male sex	2.22	1.05–4.72	0.04
BMI ≤ 34.5 kg/m^2^	2.47	1.37–4.45	0.003
Diabetes duration ≥ 11 years	2.47	1.37–4.45	0.003
MAGE ≥ 3.38 mmol/L	2.16	1.20–3.87	0.01
log MMP-3 ≥ 1.12	4.45	1.65–12.0	0.003
Diabetic retinopathy	1.97	1.09–3.57	0.02
CKD	2.81	1.51–5.23	0.001
CAD	3.68	1.74–7.77	0.001
Myocardial infarction	4.47	1.05–18.9	0.04
PAD	3.04	1.61–5.72	0.001
**Parameters associated with CAS**			
Age ≥ 66 years	3.36	2.04–5.42	<0.001
BMI ≤ 32.5 kg/m^2^	1.55	1.02–2.37	0.04
Diabetes duration ≥ 13 years	1.83	1.20–2.80	0.005
Duration of hypertension ≥ 18 years	1.95	1.26–3.04	0.003
Daily insulin dose ≥ 0.59 IU/kg	1.91	1.14–3.20	0.01
eGFR ≤ 65.5 mL/min/1.73 m^2^	1.53	1.003–2.33	0.048
log L-citrulline ≥ 2.1	4.83	1.47–15.9	0.01
Diabetic retinopathy	2.11	1.37–3.25	0.001
CKD	3.18	1.68–6.02	0.0004
CAD	2.99	1.94–4.62	<0.0001
Myocardial infarction	3.20	1.75–5.83	0.0002
PAD	2.99	1.92–4.65	<0.0001

BMI: body mass index; CA: carotid atherosclerosis; CAD: coronary artery disease; CAS: carotid artery stenosis; CI: confidence interval; CKD: chronic kidney disease; eGFR: estimated glomerular filtration rate; OR: odds ratio; PAD: peripheral artery disease; T2D: type 2 diabetes; UACR: urinary albumin-to-creatinine ratio.

**Table 5:** Parameters associated with CA and CAS in patients with T2D in multivariate logistic regression analysis.

Parameter	Adjusted OR	95% CI	*p*-Value
**Parameters associated with CA** ^1^			
Age, years	1.30	1.09–1.55	0.003
BMI, kg/m^2^	0.84	0.72–0.97	0.02
Male sex	2.91	1.08–7.81	0.03
Diabetes duration, years	0.99	0.87–1.12	0.83
MAGE, mmol/L	2.38	1.16–4.86	0.02
eGFR, mL/min/1.73 m^2^	1.06	1.003–1.13	0.04
MMP-3, ng/mL	1.09	0.9996–1.18	0.05
**Parameters associated with CAS** ^2^			
Age, years	1.19	0.96–1.49	0.12
BMI, kg/m^2^	0.60	0.39–0.90	0.01
Male sex	4.83	1.20–19.5	0.03
Diabetes duration, years	1.21	1.02–1.44	0.03
eGFR, mL/min/1.73 m^2^	1.11	1.01–1.21	0.03
L-citrulline, 10 μmol/L	1.08	1.01–1.16	0.03

Parameters of the models: ^1^ Intercept = −18.3, AUC = 0.93, KS *p*-value < 0.0001, Se = 0.84, Sp = 0.83 for L_P_ = 0.42; ^2^ Intercept = −3.08, AUC = 0.95, KS *p*-value < 0.0001, Se = 0.93, Sp = 0.87 for L_P_ = 0.54. CA: carotid atherosclerosis; CAS: carotid artery stenosis; CI: confidence interval; HbA1c: hemoglobin A1c; eGFR: estimated glomerular filtration rate; LDL: low-density lipoprotein; MAGE: mean amplitude of glycemic excursions; MMP-3: matrix metalloproteinase-3; OR: odds ratio; T2D: type 2 diabetes; UACR: urinary albumin-to-creatinine ratio.

**Table 6:** The combinations of factors associated with CA and CAS in patients with T2D.

Parameter	OR	95% CI	*p*-Value	Se	Sp
**Combinations associated with CA**					
Age ≥ 62 years AND male sex	3.20	0.96–10.6	0.06	0.16	0.94
Age ≥ 62 years AND male sex AND duration of diabetes ≥ 11 years	5.60	0.75–41.8	0.09	0.10	0.98
Age ≥ 62 years AND male sex AND BMI ≤ 34.5 kg/m^2^	7.39	1.00–54.9	0.05	0.12	0.98
(Age ≥ 62 years OR duration of diabetes ≥ 11 years) AND diabetic retinopathy	2.38	1.26–4.48	0.007	0.48	0.72
(Age ≥ 62 years OR duration of diabetes ≥ 11 years) AND CKD	3.48	1.92–6.26	0.00004	0.70	0.59
Male sex AND (age ≥ 62 years OR duration of diabetes ≥ 11 years) AND macrovascular disease (CAD OR PAD)	4.33	1.31–14.3	0.02	0.20	0.94
(Age ≥ 62 years OR duration of diabetes ≥ 11 years) AND macrovascular disease (CAD OR PAD)	3.70	2.05–6.68	0.00001	0.76	0.54
Age ≥ 62 years AND Duration of diabetes ≥ 11 years AND MAGE ≥ 3.38 mmol/L	6.11	2.15–17.4	0.001	0.33	0.93
Age ≥ 62 years AND Log MMP-3 ≥ 1.12	4.11	1.20–14.1	0.02	0.21	0.94
(Age ≥ 62 years OR duration of diabetes ≥ 11 years) AND Log MMP-3 ≥ 1.12	3.87	1.43–10.5	0.008	0.33	0.89
**Combinations associated with CAS**					
Age ≥ 66 years AND male sex	2.60	1.32–5.12	0.006	0.16	0.93
Age ≥ 66 years AND BMI ≤ 32.5 kg/m^2^	2.01	1.27–3.18	0.003	0.36	0.78
Age ≥ 66 years AND duration of diabetes ≥ 13 years	1.93	1.24–3.02	0.004	0.40	0.74
Age ≥ 66 years AND (duration of diabetes ≥ 13 years OR duration of hypertension ≥ 18 years)	2.22	1.44–3.42	0.0003	0.50	0.69
Age ≥ 66 years AND (duration of diabetes ≥ 13 years OR insulin dosage ≥ 0.59) AND diabetic retinopathy	2.24	1.39–3.61	0.001	0.35	0.80
Age ≥ 66 years AND (duration of diabetes ≥ 13 years OR insulin dosage ≥ 0.59) AND CKD	2.49	1.57–3.96	0.0001	0.47	0.74
(Age ≥ 66 years OR duration of diabetes ≥ 13 years) AND eGFR ≤ 65.5 mL/min/1.73 m^2^	2.06	1.34–3.18	0.001	0.48	0.69
Age ≥ 66 years AND (duration of diabetes ≥ 13 years OR insulin dosage ≥ 0.59) AND macrovascular disease (CAD OR PAD)	3.18	2.03–4.97	<0.00001	0.49	0.77
(Age ≥ 66 years OR duration of diabetes ≥ 13 years) AND myocardial infarction	3.67	1.86–7.24	0.0002	0.19	0.94
Age ≥ 66 years AND log L-citrulline ≥ 2.10	7.27	1.81–29.1	0.005	0.12	0.98
(Age ≥ 66 years OR duration of diabetes ≥ 13 years) AND log L-citrulline ≥ 2.10	3.86	1.50–9.94	0.005	0.26	0.92

CA: carotid atherosclerosis; CAD: coronary artery disease; CAS: carotid artery stenosis; CI: confidence interval; CKD: chronic kidney disease; HbA1c: hemoglobin A1c; eGFR: estimated glomerular filtration rate; MAGE: mean amplitude of glycemic excursions; MMP-3: matrix metalloproteinase-3; OR: odds ratio; PAD: peripheral artery disease; Se: sensitivity; Sp: specificity; T2D: type 2 diabetes; UACR: urinary albumin-to-creatinine ratio.

## Data Availability

Datasets are available from the corresponding author upon request.

## Supplementary Materials

The following are available online at https://www.mdpi.com/article/10.3390/jcm11010072/s1: Table S1: Clinical and laboratory parameters evaluated as risk factors for CA and CAS.
