# Supplementary material for: Carotid Artery Disease in Subjects with Type 2 Diabetes: Risk Factors and Biomarkers
Source: J Clin Med. 2021 Dec 24;11(1):72. doi: 10.3390/jcm11010072 (PMC8745306; doi:10.3390/jcm11010072)
Supplement: Supplementary file 1 [file jcm-11-00072-s001.zip › jcm-1486013-supplementary.pdf]

Table S1. Clinical and laboratory parameters evaluated as risk factors for CA and CAS.

	Parameter	Units/Variant
1	Age	years
2	Sex	man/woman
3	Height	cm
4	Weight	kg
5	BMI	kg/m2
6	Waist circumference	cm
7	Hip circumference	cm
8	WHR	-
9	Current smoking	no/yes
10	Diabetes duration since diagnosis	years
11	Metformin	no/yes
12	Sulphonylurea	no/yes
13	DPP-4 inhibitor	no/yes
14	GLP-1 receptor agonist	no/yes
15	SGLT2 inhibitor	no/yes
16	Insulin	no/yes
17	Insulin regimen	1: basal insulin only<br>2: premixed insulin<br>3: basal-bolus insulin treatment
18	Duration of insulin treatment	no/yes
19	Daily insulin dose	IU
20	Daily insulin dose	IU/kg
21	Diabetic retinopathy	no/yes
22	Diabetic retinopathy	1: non-proliferative<br>2: proliferative
23	CKD	no/yes
24	CKD, stage	C0-C3
25	PAD	no/yes
26	Diabetic neuropathy, peripheral	no/yes
27	Diabetic neuropathy, autonomic	no/yes
28	Valsalva maneuver ratio	-
29	Deep breathing test	bpm
30	Diabetic foot	no/yes
31	Limb amputation in medical history	no/yes
32	NAFLD	no/yes
33	Dislipidemia	no/yes
34	Dislipidemia treatment	no/yes
35	Treatment with statin(s)/fibrate(s)	1: statin(s)<br>2: fibrate(s)
36	Obesity	no/yes
37	Obesity, categories	0-3
38	Arterial hypertension	no/yes
39	Duration of arterial hypertension since diagnosis	years
40	Antihypertensive therapy	no/yes
41	Arterial hypertension, stage	0-3
42	ACE inhibitor	no/yes
43	Angiotensin II receptor blocker	no/yes
44	Diuretic(s)	no/yes
45	Beta-blocker	no/yes

46	Calcium channel blocker	no/yes
47	Centrally acting antihypertensive drug(s)	no/yes
48	CAD	no/yes
49	Angina, classes	0-4
50	Heart failure	no/yes
51	Heart failure, classes	0-4
52	Myocardial revascularizaion in medical history	no/yes
53	Myocardial infarction in medical history	no/yes
54	Stroke in medical history	no/yes
55	Antiplatelet therapy	no/yes
56	Menopause	no/yes/not applicable
57	Time since menopause	years
58	Age of menopause	years
59	HbA1c	%
60	HbA1c	mmol/mol
61	Mean glucose, fasting	mmol/L
62	Mean glucose, 2-h postprandial	mmol/L
63	Glucose, standard deviation	mmol/L
64	MAGE	mmol/L
65	HBGI	a.u.
66	LBGI	a.u.
67	Total cholesterol	mmol/L
68	LDL-cholesterol	mmol/L
69	HDL-cholesterol	mmol/L
70	Triglycerides	mmol/L
71	Uric acid	μmol/L
72	Serum creatinine	μmol/L
73	eGFR	ml/min/1.73 m2
74	Albuminuria	mg/day
75	UACR	mg/mmol
76	Proteinuria	mg/day
77	Hemoglobin	g/L
78	RBCs	$\times 10^{12}$ /L
79	WBCs	$\times 10^{9}$ /L
80	Neutrophils, abs.	$\times 10^{9}$ /L
81	Neutrophils, %	-
82	Lymphocytes, abs.	$\times 10^{9}$ /L
83	Lymphocytes, %	-
84	Neutrophil-to-lymphocyte ratio	-
85	Monocytes, abs.	$\times 10^{9}$ /L
86	Monocytes, %	-
87	Eosinophils, abs.	$\times 10^{9}$ /L
88	Eosinophils, %	-
89	Basophils, abs.	$\times 10^{9}$ /L
90	Basophils, %	-
91	Platelets	$\times 10^{9}$ /L
92	Erythrocyte sedimentation rate	mm/h
93	Fibrinogen	mmol/L
94	SFMC	mg/dL
95	D-dimer	ng/mL

BMI, body mass index; CA, carotid atherosclerosis; CAS, carotid artery stenosis; CKD, chronic kidney disease; eGFR, estimated gliomerular filtration rate; HBGI, high blood glucose index; HDL, high density lipoproteins; LBGI, low blood glucose index; LDL, low density lipoproteins; MAGE, mean amplitude of glycemic excursions; NAFLD, non-

alcoholic fatty liver disease; PAD, peripheral artery disease; SFMC, soluble fibrin monomer complex; RBCs, red blood cells; SGLT2, sodium glucose cotransporter 2; T2D, type 2 diabetes; UACR, urinary albumin-to-creatinine ratio; WBCs, white blood cells; WHR, waist-to-hip ratio.

.
